# Cosmeceutical application of extracts from the flowers, stems, and leaves of *Buddleja davidii* grown at different altitudes

**DOI:** 10.3389/fphar.2025.1551134

**Published:** 2025-05-09

**Authors:** Chih-Yu Chen, Guey-Horng Wang, Yu-Chi Chang, Singer Liu, Yueh-Te Lin, Yi-Lin Lai, Ying-Chien Chung

**Affiliations:** ^1^ Department of Tourism and Leisure, Hsing Wu University, Taipei, Taiwan; ^2^ Research Center of Natural Cosmeceuticals Engineering, Xiamen Medical College, Xiamen, China; ^3^ Department of Biological Science and Technology, China University of Science and Technology, Taipei, Taiwan; ^4^ Chill Beauty Corporation, Taipei, Taiwan; ^5^ Cancer Genome Research Center, Chang Gung Memorial Hospital at Linkou, Taoyuan, Taiwan

**Keywords:** antioxidant, antiaging, anti-inflammatory, *Buddleja davidii* Franch. [Scrophulariaceae], bioactive, cytotoxicity

## Abstract

*Buddleja davidii* Franch. [Scrophulariaceae] commonly found in the mountainous regions of Taiwan, is used as herbal medicine around the world. In spite of this*,* no research has been conducted on the physiological activities of *B. davidii* extracts from different parts of the plant and from plants grown at different altitudes. In this study, *B*. *davidii* flower, stem, and leaf extracts were prepared using distilled water, methanol, and 60% ethanol as extraction solvents. The total phenolic content of the extracts served as an indicator of their activities. Our results indicated high bioactivity in the water extract of *B*. *davidii* flowers grown at 1,500 m, the 60% ethanol extract of *B*. *davidii* stems grown at 1,000 m, and the methanol extract of *B*. *davidii* leaves grown at 1,500 m. The freeze-dried leaf extract exhibited the highest antioxidant activity, which may be attributed to its abundance of phenylethanoid glycosides and flavonoids. The major bioactive components of the flower extract were crocin, crocetin, quercetin, and rutin. Those in the stem extract were luteolin, naringenin, quercetin, acacetin, and apigenin; and in the leaf extract were verbascoside, isoverbascoside and oleanolic acid. These compounds were potentially responsible for the antiaging and anti-inflammatory activity of the flower extract (IC_50_: 28.6–125.1 mg/L), the antibacterial activity of the stem extract (minimum inhibitory concentration: 60–100 mg/L), and the antityrosinase activity of the leaf extract (IC_50_: 38.17 mg/L). For example, the antiaging activity of *B. davidii* flower extract was found to be superior to or comparable with that of the positive controls, which include EGCG (IC_50_: 67.2–162.8 mg/L), 1,10-phenanthroline (IC_50_: 46.7 mg/L), gallic acid (IC_50_: 132.6 mg/L), and tannic acid (IC_50_: 140.3 mg/L). Moreover, these extracts can be deemed safe, as they demonstrated no toxic effects on CCD-966SK, HEMn, and RAW264.7 cells at a concentration of 200 mg/L. To our knowledge, this is the first report revealing differences in activities of *B. davidii* extracts based on plant part and altitudes. The findings provide insights for potential applications of the identified bioactive compounds in health foods, herbal medicines, and cosmetics.

## 1 Introduction

Medicinal plants have long been cultivated as sources of therapeutically active compounds. Some herbal medicines even have lower side effects than conventional medicines (Nguyen and Kim, 2020). The butterfly bush (*Buddleja davidii* Franch. [Scrophulariaceae]) is a medicinal plant from the Scrophulariaceae family and is cultivated for commercial use. *B. davidii* generally grows on mountain slopes at altitudes of 800–3,000 m (Ebeling et al., 2008). The genus *Buddleja* is found in the drier subtropical regions of Asia, western Europe, southern and eastern Africa, and parts of America that lie south of the southwestern states of the United States of America. Several species have been introduced as ornamental plants because their flowers can attract butterflies. Furthermore, *B. davidii* grows widely due to its potential invasive power and adaptability (Houghton et al., 2003; Ahmad et al., 2009; Chen et al., 2020).

Previous studies have analyzed extracts from various parts of *Buddleja* and detected phenylpropanoids, flavonoids, saponins, terpenoids, sterols, and lignin (Ying and Wan, 2012). Chemical profiles of the essential oils from *Buddleja perfoliata* have been reported, and the main components included cubenol, germacrene D-4-ol, β-eudesmol, and cis-verbenol (Cuevas-Cianca et al., 2022). These bioactive compounds possess activities that are anticarcinogenic, health-promoting, wound-healing, antioxidant, antiaging, anti-inflammatory, and antimicrobial, while exhibiting low toxicity and eliciting few side effects (Wu et al., 2012; Khan et al., 2019; Nguyen and Kim, 2020; Getahun et al., 2021). The metabolites of *B. davidii* are known for their antioxidant, anti-inflammatory, and photoprotective activities (Chen et al., 2020). The flowers, stems, and leaves of *B. davidii* contain different types and concentrations of bioactive substances, including flavonoids, carotenoids, phenols, and vitamins (Ying and Wan, 2012; Diretto et al., 2021). Thus, the different parts of *B. davidii* likely contribute differently to the pharmacological and cosmeceutical activities of the plant, but these differences have not been clearly delineated yet.

The accumulation of melanin in the epidermal layer leads to melanogenesis, which is related to skin aging. Tyrosinase is the key enzyme responsible for melanin production (Mapunya et al., 2012). Furthermore, aging and skin wrinkles are often caused by the proteolytic degradation of the extracellular matrix, a process that is significantly associated with increased dermal activities of collagenase, elastase, hyaluronidase, matrix metalloproteinase-1 (MMP-1), and MMP-2 (Maity et al., 2011; Buhren et al., 2020). Some compounds derived from *B. davidii* may serve as nutritional ingredients and whitening agents in the cosmetic sector. However, the lack of a pharmacopeia of the chemical constituents of *B. davidii* extracts limits the in-depth study of the pharmaceutical and cosmeceutical potential of various parts of *B. davidii* plants grown at different altitudes. *Buddleja* plants are also cultivated in the mountainous regions of Taiwan (Chen et al., 2010). However, there have been no investigations into the impact of diverse growth environments on the pharmaceutical and cosmetic constituents found in different parts of *B. davidii*.

In this study, bioactive compounds were extracted from various parts of native *B. davidii* plants cultivated at different altitudes by using distilled water, 100% methanol, and 60% ethanol and evaluated their antioxidant, antityrosinase, antimicrobial, anti-inflammatory, and antiwrinkle properties of these extracts. The novelty of this research lies in the absence of prior literature addressing the effects of different altitudes on the biological activities of extracts. The mechanisms underlying the strong physiological effects of the extracts were elucidated by determining the compositions and amounts of phenolic acid, flavonoids, triterpenes, phenylethanoid glycosides, and other possible bioactive compounds in the extracts. Furthermore, the cytotoxicity levels of the extracts with high physiological activity were evaluated to illustrate their safety. Finally, the binding between the chemical components of *B. davidii* extracts and core enzymatic targets was evaluated through molecular docking, which is an essential method in structural molecular biology and computer-aided drug development (Jin et al., 2021).

## 2 Materials and methods

### 2.1 Plant material and extraction procedure

*Buddleja davidii* plants were collected during the autumn of 2022 from three different mountainous regions (altitudes of 1,000 m, 1,500 m, and 2,000 m) in Ren’ai Township, Nantou County, Taiwan. *B*. *davidii* was identify by a plant doctor named Bau-Yuan Hu, and voucher specimens (20221101-20221103) were submitted to the herbarium at China University of Science and Technology in Taiwan. The flowers, stems and leaves of *B. davidii* were collected, respectively.

The gathered samples were rinsed with distilled water, air-dried naturally for 2 days, pulverized to powders, sifted through a 0.5-mm mesh, stored in a drying box, and extracted with distilled water, 100% methanol, or 60% ethanol at a liquid-to-solid ratio of 10:1 (10 g sample per 100 mL solvent) in a 300-mL Erlenmeyer flask at 250 rpm for 1 h at 26^o^C. That is, each different solvent was used to extract different plant samples. The crude extracts were passed through a Whatman filter (0.45 μm), and the resulting filtrates were analyzed for total phenolic content (TPC). The extracts with high TPC were subsequently concentrated at 60°C for the aqueous extract, 50°C for the ethanolic extract, and 45°C for the methanolic extract, utilizing a rotary vacuum evaporator (Panchum Scientific Corp., Kaohsiung City, Taiwan), and the resulting residues were then subjected to lyophilization for further analysis, employing a shelf freeze dryer (Uniss Corp., Taipei City, Taiwan).

### 2.2 Microbial strains, cells, and regents

Abiding by the regulatory guidelines in the United States Pharmacopeia 51 (antimicrobial effectiveness testing) and considering the microbes associated with skin diseases, we assessed the antimicrobial effectiveness of the freeze-dried extracts from *B. davidii* flowers, stems, and leaves against various microorganisms, including *Pseudomonas aeruginosa* ATCC 9027, *Staphylococcus aureus* ATCC 6538*, Escherichia coli* ATCC 8739*, Candida albicans* ATCC 10231*, Aspergillus brasiliensis* ATCC 16404*, Cutibacterium acnes* ATCC 6919*,* and *Epidermophyton floccosum* ATCC 18397. The strains that were subjected to testing were acquired from the Bioresource Collection and Research Center (BCRC) located in Hsinchu, Taiwan. Normal HEMn cells (C-102-5C) were obtained from Cascade Biologics (Portland, OR, United States). The CCD-966SK (BCRC 60153, human skin fibroblast cell line) and RAW264.7 cells (BCRC 60001, mouse macrophagic cell line) were purchased from the BCRC. Mushroom tyrosinase (≥1,000 units/mg) along with chemicals (purity ≥99%) were purchased from Sigma-Aldrich Chemistry, located in St. Louis, United States. Lipopolysaccharide (LPS) was isolated from *E. coli* O111:B4. Proinflammatory cytokines were obtained from Invitrogen (Austria).

### 2.3 Analysis of total phenolic content and total flavonoid content

Earlier research has highlighted a favorable link between TPC, TFC, and antioxidant activity (Li et al., 2009; Muflihah et al., 2021); therefore, TPC and TFC were used as rapidly measurable indicators to screen *B. davidii* extracts. The TPC of *B. davidii* extracts was quantified in terms of gallic acid equivalents (GAE) utilizing the methodology established by Kujala et al. (2000) with slight modifications implemented. The extracts (0.5 mL) underwent oxidation using 1 mL of a 10-fold diluted Folin–Ciocalteu phenol reagent for 10 min. Subsequently, the reaction was neutralized with 1 mL of a 7.5% Na_2_CO_3_ solution and allowed to proceed for 3 h. Following this, the mixture was subjected to centrifugation at 5,000 rpm for 10 min. The absorbance of the supernatant was recorded at a wavelength of 760 nm using an ultraviolet–visible spectrophotometer (UV-2600i, Shimadzu, Japan). Gallic acid, serving as our benchmark solution, was also processed as previously outlined. The TPC was quantified either by measuring OD_760_ or by estimating it in the mg-GAE/g-dried extract, utilizing the calibration curve derived from standard solutions of gallic acid.

The total flavonoid content (TFC) was quantified utilizing the aluminum chloride colorimetric technique, and the results were reported as mg-rutin equivalents (RE)/g-dry weight (Pourmorad et al., 2006). Briefly, 100 μL of *B. davidii* extracts were mixed with 30 μL of a 5% NaNO_2_ solution for 6 min at 26^o^C. Subsequently, 30 μL of a 10% Al(NO_3_)_3_ solution was introduced to the mixture and incubated for an additional 6 min, followed by 0.4 mL of a 1 M NaOH solution and enough methanol to adjust the total volume to 1 mL. Half an hour later, we took a peek at the reaction mixture’s absorbance, capturing its essence at 500 nm with the help of an ultraviolet–visible spectrophotometer. Rutin, used as a standard solution, was also processed per the aforementioned method. The TFC was calculated from the calibration curve derived from standard solutions of rutin.

### 2.4 Evaluation of antityrosinase activity and cellular melanin levels

The antityrosinase activities of *B. davidii* extracts were evaluated utilizing the methodology established by Wu et al. (2018). The extracts derived from flower, stem, and leaf of *B. davidii* were diluted to 0–200 mg/L in dimethyl sulfoxide (DMSO) solution. Subsequently, a 30-μL sample was combined with 970 μL of phosphate-buffered saline (0.05 mM). The mixture was incorporated into a solution comprising 1 mL of L-tyrosine at a concentration of 100 mg/L and 1 mL of mushroom tyrosinase solution at an activity level of 350 U/mL and mixed homogeneously in the dark. The absorbance of the reaction mixture was assessed at a wavelength of 490 nm following a 20-min incubation period. α-Arbutin and kojic acid served as the positive control agents in the study. The half-maximal inhibitory concentration (IC_50_) is defined as the concentration of the extract that results in a 50% reduction of the initial tyrosinase activity. The antityrosinase activity of an extract is defined as the percentage of inhibition of tyrosinase, calculated using the formula provided below (Equation 1) (Wang et al., 2017).

$$\text{Tyrosinase inhibition }\left(\%\right)=\frac{\left[\left(A-B\right)-\left(C-D\right)\right]}{\left(A-B\right)}\times 100$$ (1)

where A is OD_490_ in the absence of the extract (control), B is OD_490_ without both the extract and tyrosinase (blank of A), C is OD_490_ in the presence of the extract and tyrosinase (experimental group), and D is OD_490_ in the absence of the enzyme (blank of C).

Cellular tyrosinase activity and melanin content in HEMn cells were conducted utilizing the methodology described by Wu et al. (2018). HEMn cells (2 × 10^5^ cells/well) were cultured in 24-well plates in Medium 254 supplemented with HMGS at a temperature of 37°C for a duration of 24 h in an atmosphere containing 5% CO_2_. The cells were subjected to treatment with extracts ranging from 0 to 200 mg/L for 24 h. Following this treatment, the cells were washed with phosphate-buffered saline, subsequently lysed using a cell lysis solution. The lysate was then sonicated at a cool 4°C for 10 min utilizing a Qsonica ultrasonic sonicator from Newtown, CT (United States) and centrifuged at 6,500 *g* for 15 min employing a micro-ultracentrifuge from Thermo Fisher Scientific in Waltham, MA (United States). To measure the tyrosinase activity within HEMn cells, the lysate supernatants were reacted with 2.5 mM L-3,4-dihydroxyphenylalanine for 1 h, and the OD_475_ of the solution was measured on the Epoch ELISA reader (BioTek Instruments, Santa Clara, CA, United States). In order to assess the melanin concentration in HEMn cells, the cell pellets were heated and dissolved in a solution of 1 N NaOH with 10% DMSO at a temperature of 80°C for 1 h. The OD_405_ of the solution was assessed utilizing an ELISA reader, and the melanin concentration was estimated from the OD_405_ measurement by employing a standard curve derived from synthetic melanin.

### 2.5 Cell viability assay

The viability of HEMn, CCD-966SK, and RAW264.7 cells lines was determined utilizing the 3-(4,5-dimethylthiazol-2-yl)-2,5-diphenyltetrazolium bromide (MTT) colorimetric assay, in accordance with the methodology outlined by Sittisart and Chitsomboon (2014). Briefly, 5 × 10^5^ cells were incubated overnight in each well of a 96-well plate at 37°C under 5% CO_2_. Subsequently, 0–500 mg/L of *B. davidii* extracts were incubated with the cells for 24 h. The media were removed, and 0.1 mL of 0.5 g/L MTT solution was incubated with the cells for 1 h (HEMn, CCD-966SK) or 4 h (RAW264.7). The MTT solution was removed via aspiration, and 0.1 mL of dimethyl sulfoxide was introduced to each well to facilitate the solubilization of the formazan crystals. The optical density at 570 nm (OD_570_) of the solution was assessed utilizing an ELISA reader. Cell viability was quantified as the OD_570_ value of the sample as a percentage of the OD_570_ value of the blank.

### 2.6 Analysis of scavenging activity of DPPH and ABTS

The levels of the 2,2-diphenyl-1-picrylhydrazyl (DPPH) free radical and the 2,2′-azino-bis(3-ethylbenzothiazoline-6-sulfonic acid) (ABTS) free radical are widely used indexes of the antioxidant activity exhibited by natural substances. They were conducted in accordance with the methodologies outlined by Wu et al. (2018) and Merchán-Arenas et al. (2011), respectively. Briefly, 1 mL of 0–200 mg/L *B davidii* extract in the dark. The absorbance of the mixture was assessed at a wavelength of 517 nm utilizing an ultraviolet–visible spectrophotometer, and subsequent measurements were taken following a 1-h incubation period. The DPPH scavenging activity of *B. davidii* extracts was determined using the following formula (Equation 2).

$$\text{DPPH scavenging activity }\left(\%\right)=\left(\frac{A_{0}-A}{A_{0}}\right)\times 100$$ (2)

where A_0_ represents OD_517_ measured without the extract, while A denotes OD_517_ measured in the presence of the extract.

The ABTS radical cation was produced by generated by combining a 2.45 mM K_2_S_2_O_8_ solution with a 7 mM solution of ABTS in a 1:1 ratio and allowing the mixture to react for 16 h in a dark. The ABTS solution that was prepared underwent dilution to achieve an optical density of 0.7 at a wavelength of 734 nm, and 20 μL of 0–200 mg/L *B davidii* extracts were mixed evenly with 20 μL of this solution. After an incubation of 3 min, the absorbance of this mixture was assessed at a wavelength of 734 nm utilizing an ELISA reader. Butylated hydroxytoluene (BHT) was served as a positive control for DPPH and ABTS assay. The ABTS scavenging activity of the *B. davidii* extracts was determined using the following equation (Equation 3).

$$\text{ABTS scavenging activity }\left(\%\right)=\left(\frac{A_{0}-A}{A_{0}}\right)\times 100$$ (3)

where A_0_ is OD_734_ without the extract and A is OD_734_ with the extract.

The IC_50_ values for the DPPH and ABTS scavenging activities of *B. davidii* extracts represented 50% scavenging activity (Merchán-Arenas et al., 2011).

### 2.7 Analysis of antiwrinkle activity

The activity associated with anti-wrinkle or anti-aging effects is significantly linked to the inhibition of enzymes such as collagenase, elastase, hyaluronidase, MMP-2, and MMP-1 (Wu et al., 2018). Thus, we assayed the activities of these enzymes to estimate antiwrinkle activity of the extracts. Collagenase activity was assessed utilizing a modified fluorogenic dye-quenched (DQ)-gelatin assay, as outlined by Li et al. (2020). Briefly, 10 μL of 0–300 mg/L *B davidii* extract was mixed with 10 μL of 1 U/mL collagenase and 80 μL of buffer solution in each well of a 96-well plate and reacted for 15 min. Following this, 100 μL of 15 μg/mL DQ-gelatin solution was incorporated into the mixture. The absorbance of the resulting solution was subsequently measured at an excitation wavelength of 485 nm and an emission wavelength of 528 nm using a Synergy 2 microplate reader (BioTek Instruments, Santa Clara, CA, United States) to estimate the proteolysis rate of gelatin. Elastase activity assay was conducted in accordance with the methodology outlined by Wu et al. (2018). In summary, *B. davidii* extracts were diluted with buffer solution composed of 500 mM NaCl, 100 mM HEPES, and 0.05% Tween 20 in DMSO, and this mixture was applied to each well of a 96-well plate. Subsequently, 20 μL of the diluted extracts was reacted with 50 μL of 0.171 U/mL porcine pancreatic elastase and incubated at 37°C for a duration of 10 min. Finally, 5 μL of methoxysuccinyl-Ala-Ala-Pro-Val-*p*-nitroanilide was introduced into each well, and the absorbance of the mixture was quantified at 420 nm utilizing an ELISA reader. The activity of hyaluronidase was evaluated utilizing a spectrophotometric technique (Sumantran et al., 2007). Briefly, hyaluronidase (800 U/mL), hyaluronic acid (HA) substrate (0.40 mg/mL), and *B. davidii* extracts (0–300 mg/L) were reacted at 37°C for 60 min. Hyaluronidase activity was estimated by monitoring the percentage of undigested HA substrate, which was determined by measuring the absorbance of the reaction mixture at a wavelength of 415 nm utilizing an ELISA reader. The activities of MMP-1 and MMP-2 were determined in CCD-966SK cells utilizing human MMP-1 and MMP-2 ELISA kits from RayBiotech (Norcross, GA, United States), following the methodology outlined by Chen et al. (2022). Epigallocatechin (EGCG), 1,10-phenanthroline, gallic acid, ursolic acid or tannic acid were used as positive controls in the antiwrinkle assay, if required.

### 2.8 Analysis of anti-inflammatory activity

Anti-inflammatory activity was evaluated in RAW264.7 cells. The cells were inoculated at a density of 5 × 10^5^ cells per well in a 96-well plate and subsequently incubated for 24 h. Subsequent to the renewal of the culture medium, the cells in each well were subjected to incubation with 20 μL of 0–300 mg/L *B davidii* extracts for 1 h. This was followed by the introduction of 1 μg/mL LPS to induce inflammatory responses for 24 h. The concentration of reactive oxygen species (ROS) was conducted utilizing the dichloro-dihydro-fluorescein diacetate assay, with absorbance being taken at excitation and emission wavelengths of 485 nm and 535 nm, respectively, using a Synergy 2 microplate reader (Sittisart and Chitsomboon, 2014). The concentration of nitric oxide (NO) was quantified utilizing a commercially available Griess reagent kit, with absorbance being conducted at a wavelength of 530 nm with the help of an ELISA reader and the concentration being extrapolated from a standard curve of nitrite solution (Intayoung et al., 2016). The levels of tumor necrosis factor-α (TNF-α) and interleukin-6 (IL-6) were quantified utilizing commercially available ELISA kits (R&D systems Inc., United States) in accordance with the manufacturer’s guidelines and with the help of standard curves for each cytokine (Liu et al., 2012). In anti-inflammatory study, indomethacin was served as the positive control.

### 2.9 Determination of minimum inhibitory concentration and minimum fungicidal concentration

The minimum inhibitory concentration (MIC) of *B. davidii* extracts against *E. coli*, *S. aureus*, *P. aeruginosa,* and *C*. *acnes* was assessed utilizing a tube dilution methodology (Rahman et al., 2013). Briefly, we mixed 2 mL of *B. davidii* extracts at various concentrations with 2 mL of tryptic soy broth and added a splash of 1 mL of inoculum (5 × 10^6^ cfu/mL) in a test tube. This delightful concoction was then set to incubate at 35°C. *C*. *acnes* was cultured for 48 h under anaerobic conditions, whereas the other bacterial species were cultured for 24 h under aerobic conditions. The MIC was estimated by monitoring the change in OD_600_ of the bacterial suspensions using an ultraviolet–visible spectrophotometer. The lowest concentration of *B. davidii* extracts that inhibited the observable growth of the tested bacteria was considered as the MIC.

The antifungal activity of *B. davidii* extracts was evaluated using a conventional plate count method (Chen et al., 2022). Briefly, 1 mL of *B. davidii* extracts of different concentrations were incubated with 1 mL of inoculum (5 × 10^7^ cfu/mL or spores/mL) and 100 mL of broth in a conical flask, which was then incubated at 24°C for 3 days (*C. albicans*) and a full week (*A. brasiliensis* and *E*. *floccosum*). The minimum fungicidal concentration (MFC) is defined as the lowest concentration of extracts that caused the mortality of 99.9% of the inoculated microorganisms.

### 2.10 Quantification of chemical compositions

The primary chemical compositions of *B. davidii* extracts were examined utilizing high-performance liquid chromatography (HPLC) (Hitachi, Tokyo, Japan) on a reversed-phase Econosil column (5 μm, 4.6 × 250 mm), at a flow rate of 1.0 mL/min with an injection volume of 20 µL. The chromatographic method was modified from that of Chen et al. (2022). The gradient elution protocol, utilizing solution A (deionized water) and solution B (acetonitrile) was implemented as detailed below: 0 min, 85% A; 25 min, 40% A; 50 min, 15% A; 75 min, 40% A; and 100 min, 85% A. The detection wavelength was in the range of 200–500 nm. Certain triterpenes (e.g., oleanic acid and ursolic acid) were analyzed at 210 nm (Yang et al., 2012); picrocrocin at 250 nm (Koulakiotis et al., 2015); isoflavone, alcohol, and phenolic compounds at 280 nm; phenylethanoid glycosides at 334 nm (Zhang et al., 2019); flavonoids at 350 nm (Crozier et al., 1997); and certain carotenoids (e.g., crocin and crocetin) at 440 nm. The identification of individual compounds was accomplished by comparing their retention times with those of the corresponding standards, utilizing identical experimental conditions. The concentrations of all identified compounds were quantified using calibration curves that correlate the concentrations of standard samples with their corresponding peak area values, resulting in linearity coefficients (R^2^ > 0.99).

### 2.11 Molecular docking analysis

Molecular docking calculations can provide more molecular biological information, such as the hydrogen bond force provider and the number of providers of small molecules or proteins, the polar and non-polar interaction relationships between small molecules and protein activation sites, and the configurational performance of small molecules at protein activation sites (Nwakulite et al., 2021). Molecular docking simulations were conducted on the main identified components of *B. davidii* flower, stem, and leaf extracts. The three-dimensional structures of selected chemical compounds sourced from the Human Metabolome Database, while structures of enzymes were acquired from the Protein Data Bank database. Molecular docking was conducted utilizing the iGEMDOCK 2.1 software, employing the following parameters: a population size of 300, a total of 80 generations, and a solution number of 100. The optimal docking conformation was identified as the one exhibiting the lowest energy value.

### 2.12 Statistical analysis

Data were analyzed utilizing a one-way analysis of variance, subsequently followed by Duncan’s test and expressed as the means ± standard deviations derived from at least three independent experiments. Statistically significant differences were identified with a p-value of less than 0.05. We utilized IBM SPSS, version 26 (SPSS, Chicago, IL, United States) to dive into the data.

## 3 Results and discussion

### 3.1 Effects of extraction solvent and cultivation altitudes on TPC

**FIGURE 1:**
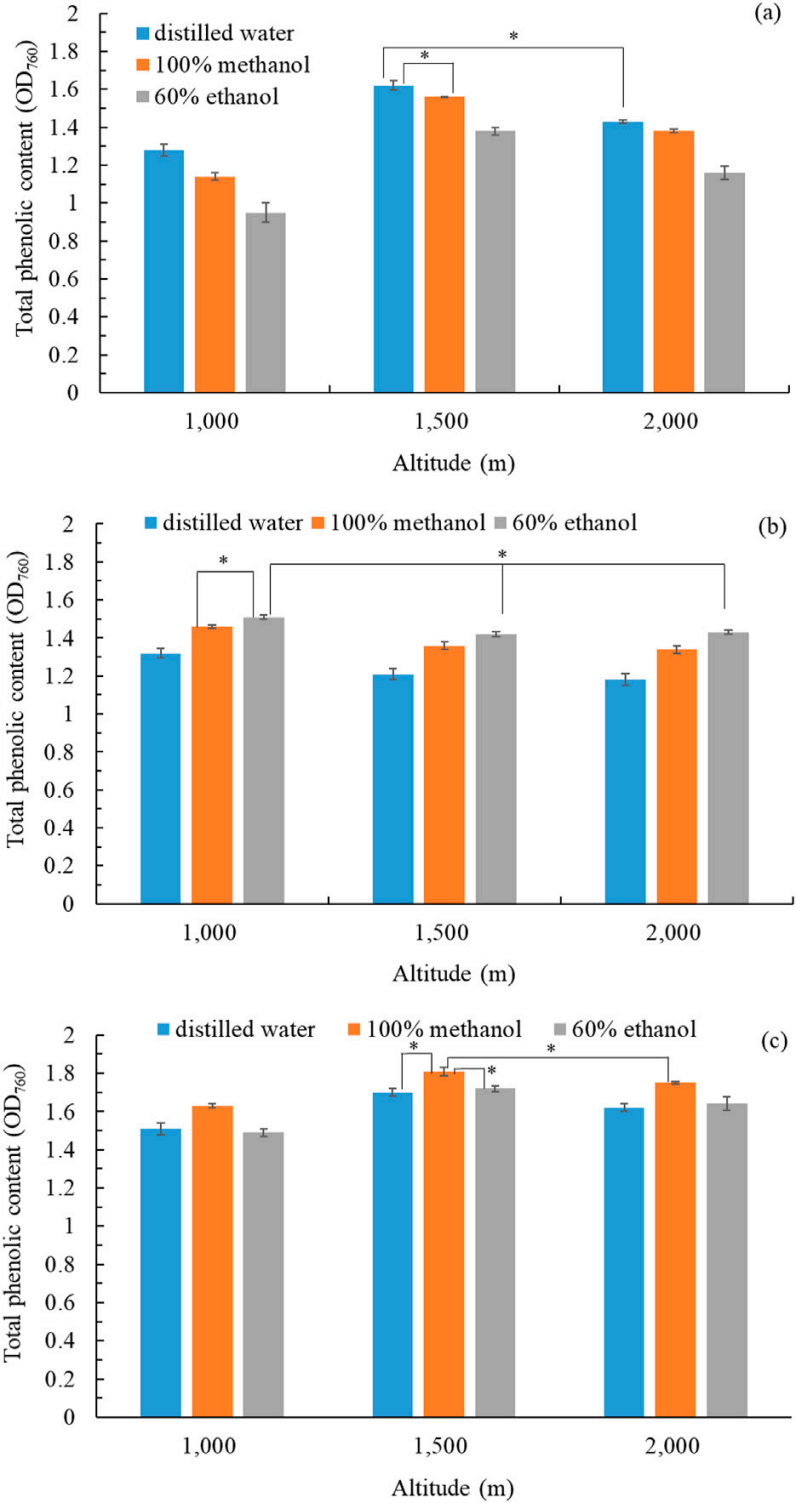
Total phenolic content in extracts: **(a)** flower extract; **(b)** stem extract; **(c)** leaf extracts of *B*. *davidii* plants collected from different altitudes and extracted using different solvents. Data are expressed as the means ± standard deviations of 3 independent experiments. Significant difference was expressed by *p < 0.05.

We used different solvents (distilled water, 100% methanol, 60% ethanol) to obtain extracts from the flowers, stems, and leaves of *B. davidii* pants cultivated at different altitudes (1,000 m, 1,500 m, 2,000 m). TPC was considered an index of the biological potency of the crude extract of *B. davidii*. The flower extract with the highest TPC (OD_760_ = 1.62 ± 0.02) was obtained using distilled water from *B. davidii* plants grown at an altitude of 1,500 m (Figure 1a). The stem extract with the highest TPC (OD_760_ = 1.51 ± 0.01) was obtained using 60% ethanol from *B. davidii* plants grown at an altitude of 1,000 m (Figure 1b). The leaf extract with the highest TPC (OD_760_ = 1.81 ± 0.02) was obtained using 100% methanol from *B. davidii* plants grown at an altitude of 1,500 m (Figure 1c). The TPC might be affected by the altitude, as indicated by the fact that herbs grown at higher altitudes tended to have higher phenolic content (Pandey et al., 2018). The increased ultraviolet radiation and lower temperatures at higher altitudes may stimulate the production of polyphenols and related bioactive compounds, which played an active role in stress response (Gülsoy et al., 2023). *B. davidii* extracts with a higher TPC likely contain more bioactive components. Zengin et al. (2015) found a positive correlation between the antioxidant activity of plant extracts and their TPC. For subsequent experiments, we used the freeze-dried water extract of *B. davidii* flowers (grown at 1,500 m), the freeze-dried ethanol extract of *B. davidii* stems (grown at 1,000 m), and the freeze-dried methanol extract of *B. davidii* leaves (grown at 1,500 m). In addition to latitude, the growth of plants at different phenological stages also influenced their physiological activity (Sharifi-Rad et al., 2020; 2022a; 2022b). By assessing the physiological traits of *B. davidii* growing at different latitudes, we chose various parts of *B. davidii* from different altitudes for potential future uses.

### 3.2 Cytotoxicity and antityrosinase activity

**FIGURE 2:**
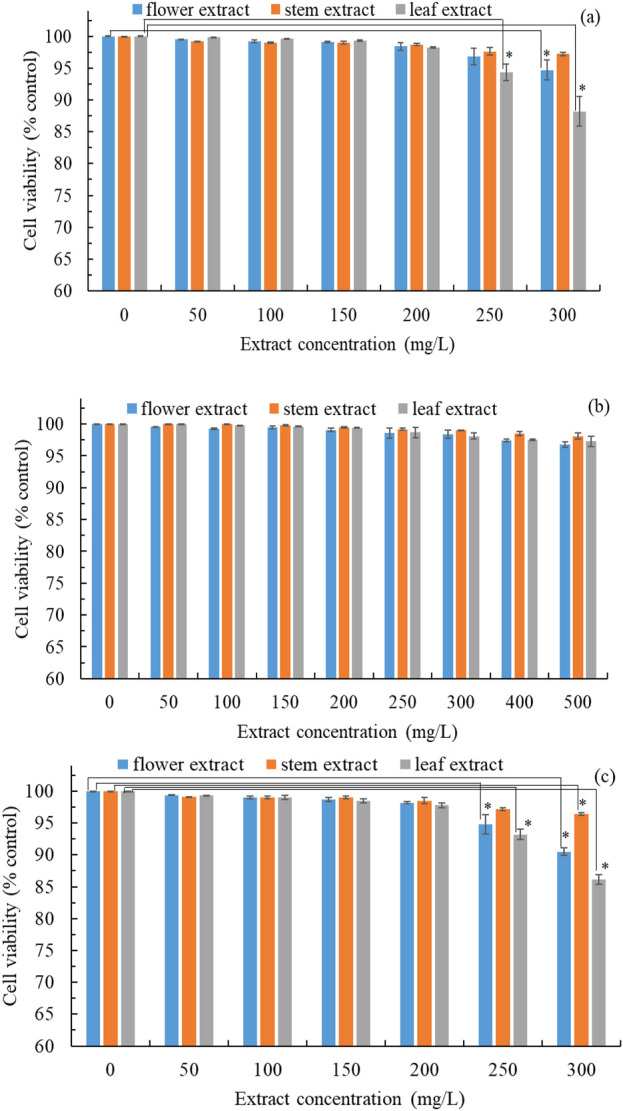
Cytotoxic effects of *B*. *davidii* extracts (flower, stem, and leaf) on **(a)** HEMn cells; **(b)** CCD-966SK cells; **(c)** RAW 264.7 cells. Data are expressed as the means ± standard deviations of 3 independent experiments. (*p < 0.05 vs blank control).

The prioritization of safety over pharmacological efficacy is of paramount importance. Therefore, we evaluated the cytotoxicity of *B. davidii* flower, stem, and leaf extracts on HEMn, CCD-966SK, and RAW264.7 cell lines utilizing the MTT assay. Cytotoxicity was assessed by comparing cell viability to the control group. HEMn cell viability was significantly reduced when treated with *B. davidii* leaf extracts at concentrations exceeding 250 mg/L (Figure 2a). The viability of CCD-966SK cells was not significantly affected by treatment with flower, stem, and leaf extracts with concentrations of 500 mg/L (Figure 2b). The viability of RAW264.7 cells was significantly affected by treatment with leaf and flower extracts with concentrations higher than 250 mg/L (Figure 2c). These findings suggest that HEMn and RAW264.7 cells were more vulnerable to *B. davidii* extracts than CCD-966SK cells. Moreover, *B. davidii* extracts from all three parts having a concentration up to 200 mg/L were safe for cells. Antityrosinase activity was assayed using extracts having concentrations less than 200 mg/L. Nguyen and Kim (2020) reported that *B. davidii* aqueous extracts exerted no significant cytotoxic effects on human HaCaT cells at concentrations reaching 100 mg/L.

**FIGURE 3:**
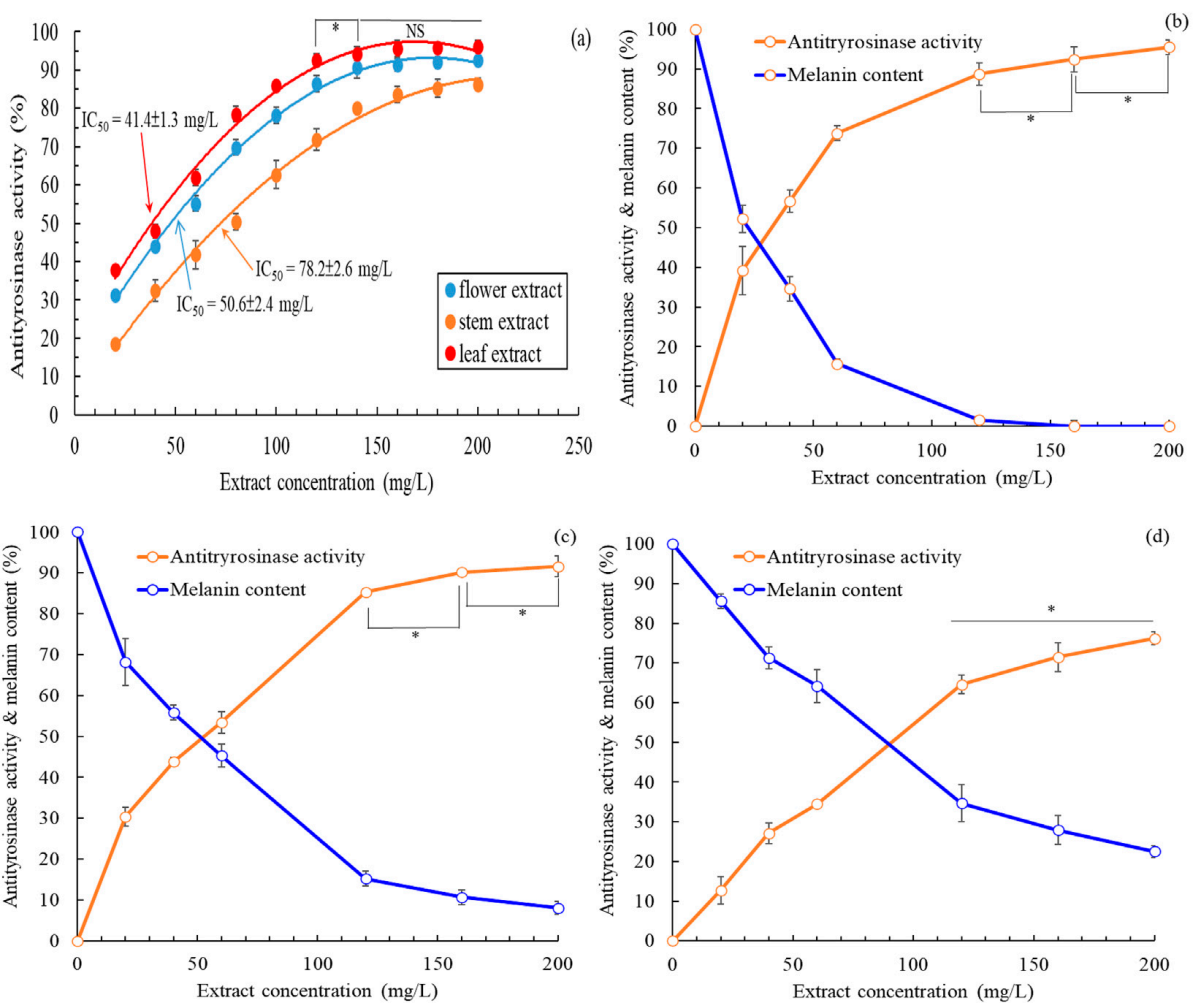
**(a)** Effects of *B*. *davidii* extracts (flower, stem, and leaf) on antityrosinase activity; **(b)** Effects on the melanin content and antityrosinase activity of HEMn cells of the **(b)** leaf extract, **(c)** flower extract, **(d)** and stem extract. Data are expressed as the means ± standard deviations of 3 independent experiments. (NS = not significant *p* > 0.05, *p < 0.05).

Tyrosinase is a crucial enzyme involved in the biosynthesis of melanin; therefore, the inhibition of tyrosinase activity can decrease melanin production and enhance skin whitening (Ho et al., 2021). The extracellular antityrosinase activity of extracts from *B. davidii* exhibited an increase in correlation with concentration (Figure 3a). A nonlinear regression analysis indicated that the IC_50_ values for the antityrosinase activity of flower, stem, and leaf extracts were 50.6 ± 2.4, 78.2 ± 2.6, and 41.4 ± 1.3 mg/L, respectively, implying that *B. davidii* leaf extracts exhibited the highest skin-whitening activity among the three extracts. The antityrosinase activity by the leaf extracts was found to be more effective than that of the positive control α-arbutin (IC_50_: 67.6 ± 3.5 mg/L) but slightly inferior to that of kojic acid (IC_50_: 32.6 mg/L). Subsequently, we evaluated the impact of different concentrations of *B. davidii* extracts on the melanin levels in HEMn cells. The melanin content exhibited a decline in correlation with the increase in extract concentration and antityrosinase activity (Figures 3b–d). A nonlinear regression analysis indicated that the IC_50_ values for the intracellular antityrosinase activity of flower, stem, and leaf extracts were 53.6 ± 2.5 mg/L, 91.8 ± 3.2 mg/L, and 38.2 ± 1.9 mg/L, respectively, which is consistent with the results obtained from the extracellular experiment.

The intracellular antityrosinase activity of *B. davidii* leaf extract was higher than its extracellular antityrosinase activity, whereas the opposite trend was observed for flower and stem extracts. The flower and stem extracts were simultaneously associated with increasing antityrosinase activity and decreasing melanin content within HEMn cells. Therefore, inhibition of tyrosinase activity was the primary mechanism underlying the inhibition of melanin production by *B. davidii* flower and stem extracts (Lim et al., 2021). However, *B. davidii* leaf extract did not display similar behavior, hinting that the underlying mechanism was more complex (e.g., modulation of the regulators of signaling pathways involving PKA/CREB, ERK, AKT, and GSK3β/β-catenin) (Kim and Hyun, 2022). Mukherjee et al. (2018) suggested that a compound may inhibit extracellular and intracellular tyrosinase to different extents because the enzyme differs in its active site oxidative state, substrate specificity, and amino acid sequence in the two locations.

### 3.3 Analysis of antioxidant and antiaging activity

The DPPH and ABTS scavenging activity assays are the preferred methods for evaluating the antioxidant activity of compounds. Table 1 presents the TPC, TFC, and the scavenging activity against DPPH and ABTS of freeze-dried *B. davidii* extracts (flowers, stems, and leaves). The DPPH scavenging activity (IC_50_: 18.3 ± 0.6 mg/L) of the leaf extract was the highest among the three extracts and was comparable to that (IC_50_: 16.3 mg/L) of the methanol extract of *Buddleja asiatica* (El-Sayed et al., 2008). The ABTS scavenging activity of the flower (IC_50_: 22.6 ± 0.9 mg/L) and leaf (IC_50_: 23.7 ± 0.2 mg/L) extracts was found to be superior to that of the stem extract. *B. davidii* leaf extracts exhibited the highest TPC and TFC among the three extracts. Higher antioxidant activities in plant extracts may suggest an abundance of phenolic compounds or carotene derivatives (Mahlke et al., 2009), with additional evidence provided in the results of Section 3.6. The DPPH and ABTS radical scavenging activities exhibited by all three *B. davidii* extracts were found to be more effective than those of the positive control BHT (IC_50_: 58.1 ± 1.2 mg/L and 50.6 ± 0.37 mg/L, respectively).

**TABLE 1:** Antioxidant properties of freeze-dried extracts from different parts of *Buddleja davidii.*

Extracts	DPPH (IC_50_: mg/L)	ABTS (IC_50_: mg/L)	Total phenolic content (mg-GAE/g-dry weight)	Total flavonoid content (mg-rutin/g-dry weight)
Flower extract	56.4 ± 0.7^a^	22.6 ± 0.9^a^	82.6 ± 0.8^a^	116.7 ± 2.5^a^
Stem extract	27.8 ± 0.1^b^	34.9 ± 0.6^b^	105.4 ± 1.7^b^	135.1 ± 3.2^b^
Leaf extract	18.3 ± 0.6^c^	23.7 ± 0.2^a^	136.4 ± 2.1^c^	183.5 ± 2.9^c^

Within each column, different superscript letters (a–c) indicate statistically different values according to Duncan’s test at *p* < 0.05.

To maintain vibrant and radiant skin, the inhibition of collagenase, elastase, hyaluronidase, MMP-1, and MMP-2 within the skin is crucial. Table 2 presents the antiaging activity of freeze-dried extracts from different parts of *B. davidii*. *B. davidii* flower extract exhibited the best antiaging activity, with IC_50_ values for inhibiting aging-related enzymes in the range of 62.7–125.1 mg/L, followed by the leaf and stem extracts. *B. davidii* flower extract presented better antiaging activity than some positive controls, such as EGCG (IC_50_: 90.6 ± 3.7 mg/L, 67.2 ± 1.4 mg/L, and 162.8 ± 7.1 mg/L for anti-MMP-1, anti-MMP-2, and antielastase activities, respectively), 1,10-phenanthroline (IC_50_: 46.7 ± 0.5 mg/L for anticollagenase activity), gallic acid (IC_50_: 132.6 ± 10.3 mg/L for anticollagenase activity), ursolic acid (IC_50_: 32.8 ± 4.2 mg/L for antielastase activity), and tannic acid (IC_50_: 140.3 ± 11.6 mg/L for antihyaluronidase activity). *Buddleja officinalis* (a close relative of *B. davidii*) has been shown to inhibit the aging-related enzymes elastase and MMPs (Kucukguven and Khalil, 2013; Chen et al., 2023).

**TABLE 2:** Antiaging activity (IC_50_, mg/L) of freeze-dried extracts from different parts of *Buddleja davidii.*

Extracts	MMP-1 activity	MMP-2 activity	Collagenase activity	Elastase activity	Hyaluronidase activity
Flower extract	103.5 ± 1.7^a^	125.1 ± 1.7^a^	62.7 ± 0.5^a^	90.5 ± 0.7^a^	87.2 ± 0.4^a^
Stem extract	216.7 ± 2.1^b^	234.5 ± 3.0^b^	207.4 ± 4.5^b^	186.4 ± 1.7^b^	201.7 ± 4.1^b^
Leaf extract	182.4 ± 2.4^c^	197.2 ± 2.9^c^	114.6 ± 0.9^c^	134.9 ± 1.4^c^	126.5 ± 1.0^c^

Within each column, different superscript letters (a–c) indicate statistically different values according to Duncan’s test at *p* < 0.05.

### 3.4 Examination of anti-inflammatory activity

**FIGURE 4:**
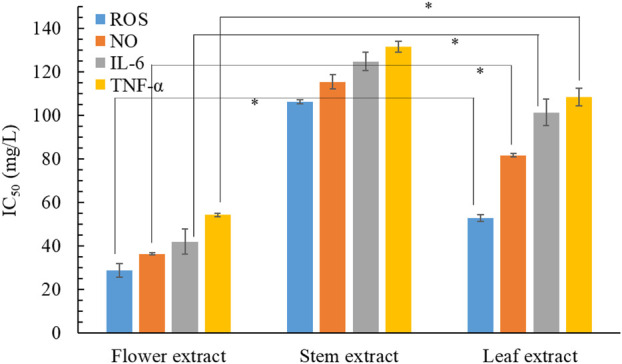
Effects of *B*. *davidii* extracts (flower, stem, and leaf) on the production of NO, ROS, IL-6, and TNF-α in LPS-stimulated RAW264.7 cells. Significant difference was expressed by *p < 0.05.

The overproduction of proinflammatory mediator by macrophages can result in various inflammatory diseases. Therefore, one effective therapeutic strategy involves lowering the levels of inflammatory mediators and cytokines. Figure 4 shows the anti-inflammatory activities of the 3 *B. davidii* extracts, expressed as IC_50_ values for the inhibition of various inflammatory mediators and cytokines. The flower extract had the lowest IC_50_ value among all three extracts for the inhibition of ROS, NO, and TNF-α, implying that it possessed the strongest anti-inflammatory activity, with the leaf and stem extracts showing comparatively lesser effects. Specifically, the anti-inflammatory activity of the flower extract was 2.4–3.7 times more effective than that of the stem extract and 1.8 to 2.4 times more effective than that of the leaf extract. The IC_50_ of *B. davidii* flower extract for inhibiting TNF-α production (54.2 ± 0.8 mg/L) was inferior to those of cyclocitralosides A and B, pure compounds isolated from *B. officinalis* flower extract (IC_50_: 6.83–7.71 mg/L; Huang et al., 2021), and the positive control indomethacin (a potent nonsteroidal anti-inflammatory drug) (IC_50_: 51.4 ± 1.2 mg/L). Furthermore, Nguyen and Kim (2020) reported that *B. davidii* aqueous extract exerted the strongest inhibitory effect on the inflammatory processes associated with *C. acnes.* A higher concentration of flavonoids in the leaf extract of *Buddleja scordioides* was associated with high anti-inflammatory activity (Macías-Cortés et al., 2022). These results clearly illustrate the utility of using *B. davidii* extracts, especially the flower extract, as a natural anti-inflammatory ingredient for skin care.

### 3.5 Analysis of antimicrobial activity

Wu et al. (2018) reported that antimicrobial activity is positively correlated with antioxidant activity. Table 3 lists the antimicrobial characteristics of freeze-dried extracts from different parts of *B. davidii*. The stem extract of *B. davidii* demonstrated the most significant antimicrobial activity, with the leaf and flower extracts exhibiting comparatively lower levels of efficacy. Pendota et al. (2013) discovered that *Buddleja salviifolia* leaf extract possessed antimicrobial properties that resulted from additive effects or synergistic interactions between the active compounds in the extract. In the present investigation, the MIC of *B. davidii* stem extract against *S. aureus*, *E. coli*, *P. aeruginosa,* and *C. acnes* was determined to be between 60 and 100 mg/L. This efficacy is notably higher than that observed for a hexane extract of *Buddleja perfoliate*, which exhibited an MIC of 200 mg/L (Cuevas-Cianca et al., 2022). The MFC of *B. davidii* stem extract against *C. albicans*, *A. brasiliensis,* and *E. flocosum* (tinea pedis related pathogen) was in the range of 30–50 mg/L, which was superior to that of chloroform extracts of *Buddleja globosa* (MFC: 100 mg/L) and *B. davidii* stembark (MFC: 62.5 mg/L) (Houghton et al., 2003). Given that it is safe to apply to the skin at a dose between 30 and 100 mg/L (Figure 2), *B. davidii* stem extract can potentially be used in cosmetic products whose purpose is to clean, sterilize, and preserve the skin.

**TABLE 3:** Minimum inhibitory concentration (MIC) and minimum fungicidal concentration (MFC) of freeze-dried extracts from different parts of *Buddleja davidii.*

Extracts	MIC (mg/L)			MFC (mg/L)			
	*S. aureus*	*E. coli*	*P. aeruginosa*	*C*. *acnes*	*C. albicans*	*A. brasiliensis*	*E*. *flocosum*
Flower extract	1000^a^	800^a^	500^a^	300^a^	1500^a^	2000^a^	2000^a^
Stem extract	100^b^	80^b^	80^b^	60^b^	30^b^	50^b^	50^b^
Leaf extract	250^c^	200^c^	200^c^	150^c^	150^c^	200^c^	250^c^

Within each column, different superscript letters (a–c) indicate statistically different values according to Duncan’s test at *p* < 0.05.

### 3.6 Identification of bioactive compounds

Table 4 lists the major chemical components (relative concentrations >0.5%) detected in the three *B. davidii* extracts. A total of 21, 16, and 21 major active components were detected using HPLC in the flower, stem, and leaf extracts, respectively (Table 4). In order of decreasing abundance, the flower extract primarily contained crocin, crocetin, quercetin, sinapic acid, rutin, and picrocrocin. Crocin, crocetin, and picrocrocin are carotenoids that have anti-inflammatory and antiaging activities (Roniawati et al., 2021; Roshanravan and Ghaffari, 2022). Moreover, the flower extract contained sinapic acid, a bioactive phenolic acid with anti-inflammatory and antiaging activities (Chen, 2016), and rutin, a flavonoid with antiaging activity (Choi et al., 2016). The presence of these components might explain why *B. davidii* flower extract exhibited better anti-inflammatory and antiaging activities than the other two extracts. The major components of the stem extract were luteolin, oleanolic acid, naringenin, quercetin, acacetin, and apigenin. Luteolin, quercetin, acacetin, naringenin, and apigenin are flavonoids that possess antimicrobial activities (Zhou et al., 2014; Ayeleso et al., 2017; Aghababaei and Hadidi, 2023), which can explain why *B. davidii* stem extract exerted higher antimicrobial activities than the other two extracts. The primary constituents identified in the leaf extract were oleanolic acid, verbascoside, luteolin, isoverbascoside, luteolin-7-O-β-D-glucoside, ursolic acid, apigenin, and martynoside, similar to the composition of an 80% methanol extract of *B. polystachya* leaves (Getahun et al., 2021). Verbascoside, isoverbascoside, and martynoside are phenylethanoid glycosides; oleanolic acid and ursolic acid are triterpenes; and luteolin, luteolin-7-O-β-D-glucoside, and apigenin are flavonoids, all of which were detected in *B. davidii* leaf extract. Burger et al. (2017) proposed that luteolin and apigenin can serve as skin whitening agents. Moreover, martynoside has been shown to exert considerable skin whitening activity (Muñoz et al., 2013), and verbascoside, isoverbascoside, oleanolic acid, and ursolic acid offer photoprotection against ultraviolet radiation to prevent melanin production (Díaz-Barradas et al., 2016; Gómez-Hernández et al., 2021), which can explain why *B. davidii* leaf extract exhibited better skin whitening activity than the other two extracts. Even so, different compounds within the extracts may have potential synergistic effects on their physiological activity, which will be investigated in future studies.

**TABLE 4:** Chemical compositions and contents of extracts from various parts of *Buddleja davidii*.

Chemical composition	Retention times^a^ (min)	Flowers extract	Stem extract	Leaf extract
		Relative content		
Picrocrocin	15.29	5.48%	—	—
Caffeic acid	19.00	4.15%	4.06%	3.04%
Verbascoside	20.43	—	—	10.25%
Isoverbascoside	21.67	—	—	7.24%
p-coumaric acid	23.06	3.19%	5.31%	2.14%
Ferulic acid	24.02	3.25%	3.71%	4.12%
Sinapic acid	25.17	7.12%	2.51%	2.28%
Rutin	27.16	6.24%	6.90%	3.74%
Luteolin-7-O-β-D-glucoside	27.53	0.79%	4.56%	7.20%
Apigenin-7-O-β-D-glucoside	30.61	1.27%	4.81%	2.57%
Linarin	31.50	—	—	3.60%
Quercetin	35.89	9.21%	8.74%	3.64%
Naringenin	36.77	2.38%	9.35%	4.01%
Hesperetin	39.35	4.85%	—	1.27%
Luteolin	39.38	1.21%	14.31%	9.18%
Oleanolic acid	40.31	2.87%	9.64%	12.43%
Crocin	41.28	23.54%	—	—
Ursolic acid	41.52	—	—	7.04%
Apigenin	42.51	1.60%	7.48%	6.01%
Crocetin	43.72	14.65%	—	—
Acacetin	45.62	1.15%	8.60%	2.48%
Martynoside	47.18	2.13%	4.27%	5.44%
Jionoside D	48.14	1.45%	—	1.68%
Angoroside C	50.07	2.85%	2.91%	—
Campneoside II	52.47	0.62%	2.84%	0.64%
Total		100%	100%	100%

^a^ The chromatographic method was modified from that of Chen et al. (2022). The gradient elution protocol, utilizing solution A (deionized water) and solution B (acetonitrile) was implemented as detailed below: 0 min, 85% A; 25 min, 40% A; 50 min, 15% A; 75 min, 40% A; and 100 min, 85% A. The detection wavelength was 280 nm. The individual compounds were quantified at specific wavelengths, as detailed in Section 2.10.

### 3.7 Molecular docking analysis

Molecular ducking study could help understand the binding mode and binding interactions between the inhibitory compounds and the target site. We performed molecular docking to explore the binding interactions between the predominant bioactive compounds and the active sites of target enzymes selected based on the physiological activities of these compounds. We especially focused on research on antiaging and antityrosinase activities. *B. davidii* flower extract possessed the best antiaging activity among the three extracts (Table 2), so we subjected the major chemical components of *B. davidii* flower extract: crocin, crocetin, quercetin, sinapic acid, picrocrocin, and rutin to molecular docking with aging-related enzymes (Table 5). The findings indicated that the binding affinities of each of the different compounds for any given enzyme differed from each other. A reduced binding energy signifies a higher affinity of the small molecule ligand for the catalytic site (Xu et al., 2024). Crocin exhibited the best binding energies against MMP-2 (PDB code: 8H78) and hyaluronidase (PDB code: 2PE4) (−133.34 and −167.41 kcal/mol, respectively). Crocetin exhibited the best binding energy against elastase (PDB code: 1FLE) (−140.17 kcal/mol). Quercetin had the best binding energy against MMP-1 (PDB code: 3SHI) (−148.21 kcal/mol), whereas rutin had the best binding energy against collagenase (PDB code: 2CLT) (−135.37 kcal/mol). Bastani et al. (2022) proposed that the potent antioxidant activity of crocin may inhibit the activities of MMP-2 and hyaluronidase. Bari et al. (2023) reported that crocetin could confer protection from oxidative stress-induced damage through its antielastase activity. In addition, quercetin could downregulate MMP-1 expression (Beken et al., 2020), and rutin exhibited good antiaging activity (Phumat et al., 2023).

**TABLE 5:** Results of molecular docking analysis of crocin, crocetin, quercetin, sinapic acid, picrocrocin, and rutin.

Target enzyme	Crocin	Crocetin	Quercetin	Sinapic acid	Picrocrocin	Rutin
	Total energy (kcal/mol)					
MMP-1	−120.93	−109.26	−148.21	−74.37	−101.33	−127.77
MMP-2	−133.34	−86.09	−97.54	−81.33	−103.78	−115.53
Collagenase	−109.45	−92.51	−103.06	−82.82	−88.71	−135.37
Elastase	−131.09	−140.17	−97.63	−80.70	−97.69	−128.48
Hyaluronidase	−167.41	−104.97	−103.25	−81.97	−92.56	−130.76

*B. davidii* leaf extract exhibited the best antityrosinase activity among the three extracts (Figure 3), so its major ingredients were docked against tyrosinase. The binding energies of oleanolic acid, verbascoside, luteolin, isoverbascoside, ursolic acid, and apigenin to tyrosinase (PDB code: 5M8L) were −120.88, −136.86, −101.54, −112.07, −86.29, and −95.93 kcal/mol, respectively. Verbascoside exhibited the best binding energy to tyrosinase. Matos et al. (2022) reported that verbascoside showed good tyrosinase inhibition activity.

**FIGURE 5:**
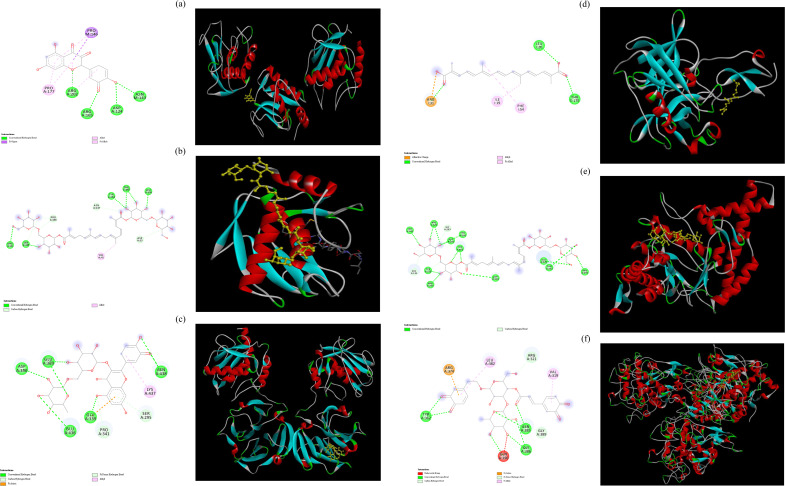
Molecular docking interactions and docking complex of the optimal phytochemical compounds against tested enzymes. **(a)** molecular docking of the interactions between quercetin and MMP-1; **(b)** molecular docking of the interactions between crocin and MMP-2; **(c)** molecular docking of the interactions between rutin and collagenase; **(d)** molecular docking of the interactions between crocetin and elastase; **(e)** molecular docking of the interactions between crocin and hyaluronidase. **(f)** molecular docking of the interactions between verbascoside and tyrosinase.

Among the various compounds examined, quercetin exhibited the most favorable docking interactions with MMP-1, facilitated by the formation of a π–alkyl bond with Pro177, a π–sigma bond with Pro146, and H-bonds with Arg165, Arg202, Asp124, and Asn143 (Figure 5a). Crocin exhibited the best docking interactions with MMP-2, facilitated by the formation of an alkyl bond with Val41, C−H bonds with Pro166, Asn147, and Asp33, and H-bonds with Arg49, Ser165, Tyr144, Thr145, and Leu106 (Figure 5b). Rutin demonstrated the most favorable docking interactions with collagenase, facilitated by the establishment of an alkyl bond with Lys437, C−H bonds with Pro341 and Ser295, and H-bonds with Asp390, Glu389, Glu436, Glu339, and Asn438 (Figure 5c). Crocetin demonstrated the most favorable docking interactions with elastase, which were attributed to the establishment of an ionic bond with Arg31, π–alkyl bonds with Ile19 and Phe54, and H-bonds with Leu20 and Thr175 (Figure 5d). Crocin demonstrated optimal docking interactions with hyaluronidase, facilitated by the formation of C−H bonds with residues Glu131 and Gly203, as well as H-bonds with Trp130, Arg134, Asp206, Tyr202, Tyr208, Tyr210, Arg265, Asp292, Glu325, Thr293, and Asn295 (Figure 5e). Verbascoside demonstrated the most favorable docking interactions with tyrosinase, facilitated by the formation of a π–carbon bond with Arg374, an unfavorable bump with Gln390, C−H interactions with Arg321 and Gly389, π–alkyl interactions with Leu382 and Val319, as well as H-bonds with Tyr362, Asn385, and Gly386 (Figure 5f). Subbaiyan et al. (2020) indicated that these H-bonds played a central role in maintaining the structural stability of the protein–ligand complex. Conversely, unfavorable bump interactions revealed poor binding between amino acids and ligands (Zainab et al., 2020). Based on the experimental and molecular docking results, we conclude that the main ingredients of *B. davidii* flower and leaf extracts are responsible for their observed antityrosinase and antiaging properties.

## 4 Conclusion

In the present study, we demonstrated that *B. davidii* extracts contained phenylethanoid glycosides, flavonoids, phenolic acids, carotenoids, and triterpenes. The extracts from all three plant parts possessed potent antioxidant, antiaging, antimicrobial, or/and anti-inflammatory activities. Antiaging/anti-inflammatory activity was high in the flower extract, antimicrobial activity was high in the stem extract, and skin-whitening activity was high in the leaf extract. Molecular docking analysis further suggested that crocin, crocetin, quercetin, and rutin in the flower extract were responsible for its antiaging activity, whereas verbascoside in the leaf extract was responsible for its antityrosinase activity. In conclusion, this serves as a foundation for future studies exploring the physiological properties of *B. davidii* extracts and their bioactive compounds. Products containing *B. davidii* extracts can be developed for use in cosmetics, health products, medicine, and clinical studies. Thus, we select various parts of *B. davidii* from different altitudes for utilization. *B. davidii* cultivated in Taiwan shows promise for several practical applications. The underlying mechanisms of action will need to be investigated further in the future.

## Data Availability

The original contributions presented in the study are included in the article/supplementary material, further inquiries can be directed to the corresponding author.
